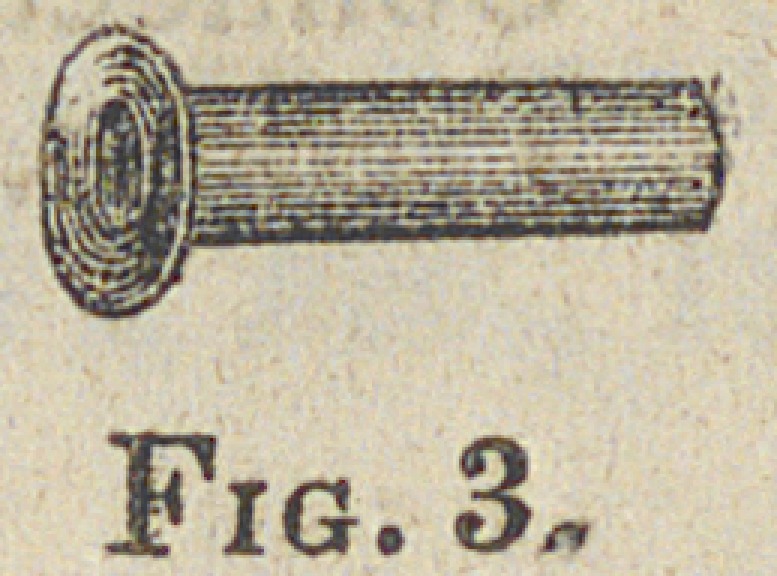# Dr. T. W. Baxter’s Combined Bellows, Blow-Pipe, and Forge

**Published:** 1854-04

**Authors:** 


### *Dr. T. W. Baxter's Combined Bellows, Blow-Pipe, and Forge.*

A cheap and useful article for the dentist's laboratory. The blast is regular, and of sufficient force for any purpose the dentist may wish. By examining the following engraving, the construction will be easily understood. This is so constructed, that it can be attached to a table or work-bench, and easily removed.

The forge is a foot and a half high, and one foot in diameter, and is composed of sheet iron with a twelve-inch door, and three-inch smoke pipe, and the bottom is filled in with clay.

The bellows conssits of a keg sawed off at the first hoop from the bulge, a head fitted in eight inches from the top, and in it a valve, four inches in diameter; at the lower head where the treddle is attached, there is another valve of the same size. This upper and lower head moves down and up near the center partition of the keg; the leather being six inches wide, gives each head twelve inches play horizontal. This keg is fastened to the table by three rods, which also answer for guides to the upper head, which is fastened down with spiral springs or weights, and the lower one the same.

Fig. 1. Forge.

" 2. Leather portion of Bellows.

" 3. Body composed of a keg.

" 4. Connecting Pipe.

" 5. Flexible Tube and Blow-Pipe.

6. Connecting Rods.

7. Treddle.

The treddle is attached to the lower head with a bolt screwed in the head, and then with the lower end of the bolt flattened, and a hole punched through; this gives it sufficient play. A screw is then passed through the treddle into the table-leg; with this down, and your leather attached to the keg and the upper and lower pieces, we have the old tub bellows in miniature.

The connecting pipe, is a tin tube, one inch in diameter, fastens to the bellows by means of a tube of copper screwed in the bellows and to the blast furnace, by being pressed in the outer end of the iron nozzle, which is embedded in the keg to near its point, when it turns up, to prevent blowing the sparks out of the door, when melting gold.

*Fig. 2.—Baxter's Riveting Forceps.*

The accompanying engraving represents this instrument full size. I use it for riveting the backings on the teeth. One motion of the hand does the work, and leaves a smooth surface ready for the solder; and the expansion of the platinum by heat gives plenty of room for the solder to pass around the pins. By the use of this, there is no danger of breaking the teeth, or loosening the pins.

Fig. 2.

Fig. 3.

*Fig. 3.—Baxter's Artificial Leech.*

The engraving of this is full size. This is a useful article for the dentist, as well as the physician. This leech is constructed of a silver tube, with a flange at one end, as large as is required, and of different shapes; the balance is a common Britannia syringe, with a small brass spiral spring, either on the inside, or around the piston, of sufficient strength to force the piston out. You then have the motive power for your leech; put the small tube on the nozzle of the syringe; make your leech bite with your spear-point, or locked flat drill; you then force down the piston as far as the spring will allow, and it is ready for use.

## Figures and Tables

**Fig. 1. f1:**
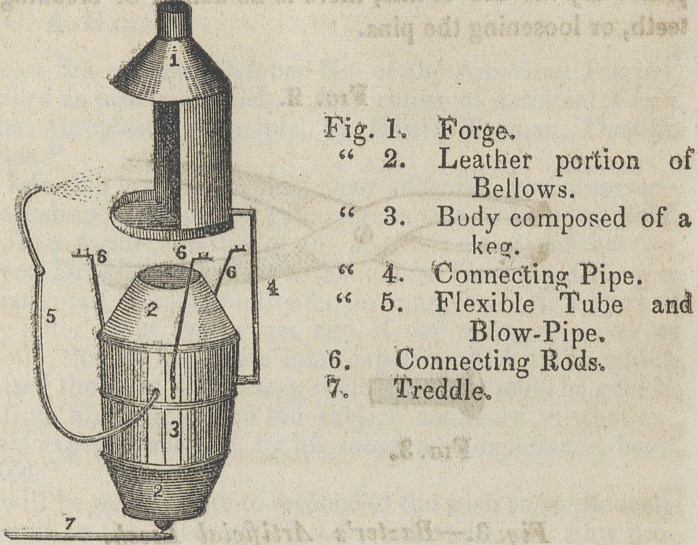


**Fig. 2. f2:**
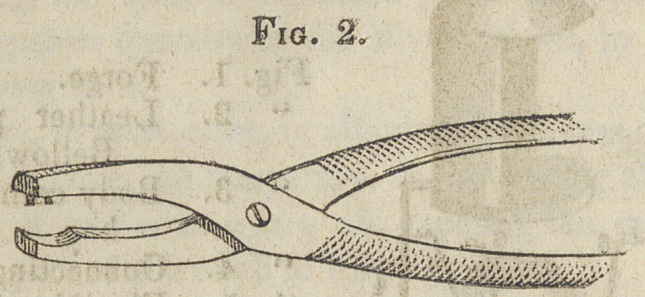


**Fig. 3. f3:**